# Giant left atrial myxoma causing mitral regurgitation through annulus dilatation

**DOI:** 10.1002/ccr3.7742

**Published:** 2023-07-30

**Authors:** Danfeng Xu, Xuejie Li

**Affiliations:** ^1^ Department of Anesthesiology, West China Hospital Sichuan University and The Research Units of West China (2018RU012), Chinese Academy of Medical Sciences Chengdu China

**Keywords:** left atrial myxoma, mitral annuloplasty, mitral annulus dilatation, mitral regurgitation

## Abstract

**Key Clinical Message:**

Giant left atrial myxoma causing mitral regurgitation through mitral annulus dilatation without affecting leaflet function is rarely reported.

**Abstract:**

This is a case of a 37‐year‐old man suffering from worsening exertional dyspnea detected left atrial myxoma 92 × 43 mm. Radical mass resection and mitral annuloplasty were performed simultaneously. Giant left atrial myxoma causing mitral regurgitation through mitral annulus dilatation without affecting leaflet function is rarely reported.

## 1 CASE PRESENTATION

**FIGURE 1:**
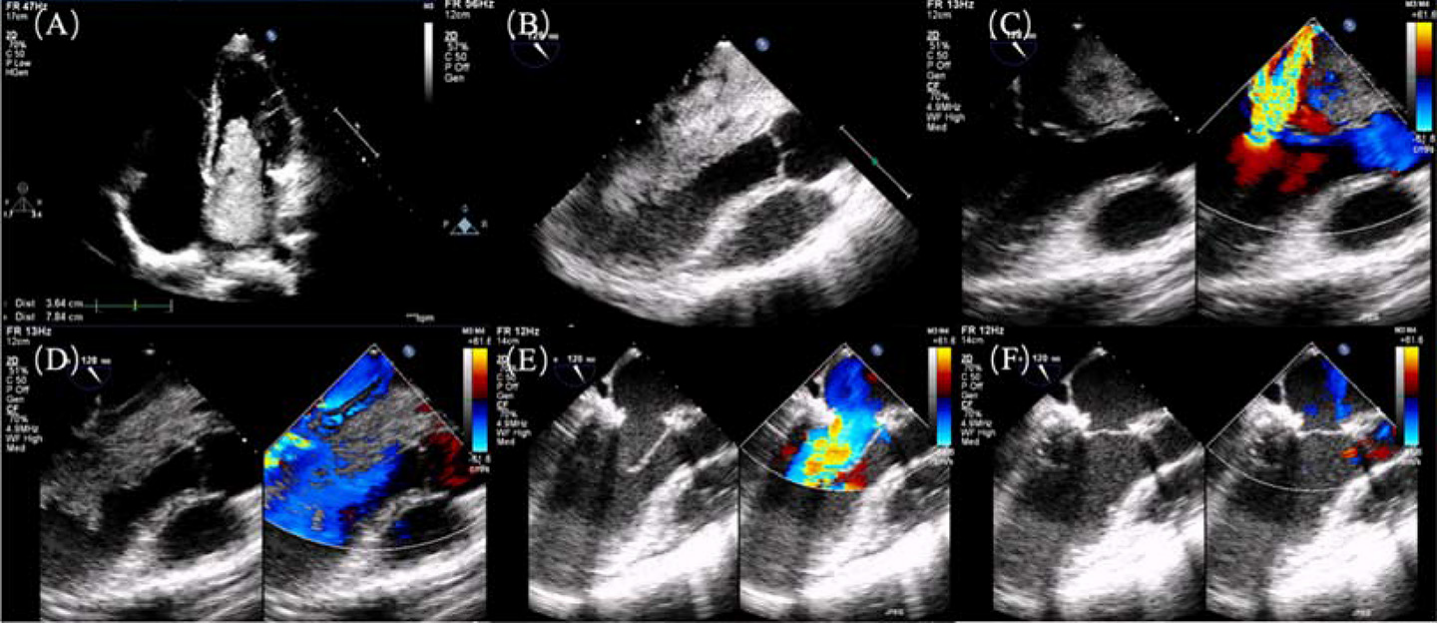
(A) Preoperative echocardiography showed the size of the left atrial myxoma was 92 × 43 mm, which was the occlusion of the mitral valve orifice. (B) ME LAX view showed that the left atrial myxoma occupied almost the entire left ventricular inflow tract during diastole and mitral valve. (C) ME LAX view showed left atrial myxoma pushed into the left atrium as a whole during systole and with severe mitral regurgitation. (D) ME LAX view showed the atrial myxoma was obstructing the left ventricular inflow tract, causing inflow tract obstruction during diastole. (E) After mitral valve annuloplasty, the mitral valve opened well during diastole. (F) There was no mitral regurgitation after mitral valve annuloplasty.

A 37‐year‐old man, without medical history or cardiovascular risk factors, was admitted with exercise‐related syncope and dyspnea which started the previous month. He did not have any embolic events preoperation. Transthoracic echocardiogram (Figure 1A) revealed a 92 × 43 mm mass in the left atrium, causing severe functional mitral stenosis and mitral regurgitation. The mass was prolapsing into the left ventricle during diastole. Cardiac surgery under cardiopulmonary bypass was planned. The hemodynamics was instable during induction due to large tumor. Intraoperative transesophageal echocardiography (TEE) examination was performed after induction of general anesthesia. It showed that the left atrial myxoma occupied almost the entire left ventricular inflow tract during diastole in ME LAX (mid‐esophageal long axis) view (Figure 1B). Left atrial myxoma pushed into the left atrium as a whole during systole, and with severe mitral regurgitation (Figure 1C). The left atrial myxoma was obstructing the left ventricular inflow tract, causing inflow tract obstruction during diastole (Figure 1D). After mitral valve annuloplasty, the mitral valve opened well during diastole (Figure 1E), and the mitral valve closed completely with no mitral regurgitation (Figure 1F). The postoperative period was uneventful. At discharge, the transthoracic echocardiogram revealed no mitral regurgitation or stenosis, and the definite anatomopathological exam confirmed the diagnosis of a cardiac myxoma.

A mitral valve inflow obstruction caused by an LA myxoma represents an important hemodynamic consequence. Myxoma disturbs LV inflow and induces elevated LA pressure and pulmonary hypertension. In this case, if the myxoma blocked the main flow, the goal of hemodynamic management is similar to that of MS (mitral stenosis) management. Maintaining myocardial contractibility, preloads, and afterloads is important; keeping the heart rate stable is also essential.

Radical resection is sufficiently effective for typical left atrial myxoma. Additional procedures sometimes are required in tumors that reached the valve leaf left or are accompanied by chordal dysfunction.^1^ Mitral annulus dilatation due to the tumor is uncommon^2^ and considered to be caused functionally by left atrial dilatation due to mitral stenosis or direct mechanical compression from the large myxoma. Myxomas might camouflage an easily hidden mitral regurgitation caused by annular dilatation other than the one generated by a prolapse of mitral leaflets. In this patient, the myxoma had completely exited into the left atrium during systole without squeezing the mitral valve leaflets, so the mechanism of mitral regurgitation is annular dilation. Therefore, mitral valve annuloplasty was performed at the time of myxoma resection simultaneously, avoiding the second cardiopulmonary bypass.

## AUTHOR CONTRIBUTIONS

**Danfeng Xu:** Conceptualization; data curation; investigation; methodology; project administration; visualization; writing – original draft. **Xuejie Li:** Conceptualization; data curation; methodology; writing – review and editing.

## CONFLICT OF INTEREST STATEMENT

There is no conflict of interest.

## ETHICS STATEMENT

Not applicable.

## CONSENT

Written informed consent was obtained from the patient to publish this report in accordance with the journal's patient consent policy.

## Data Availability

We documented the patient's data reported in the article. We will share the de‐identified data on reasonable request. To request the data, please contact the corresponding author.

## References

[1] Yu Q , Chen F , Chen L . Clinical value of echocardiography in diagnosing left atrial myxoma combined with mitral chordae tendineae rupture. Int J Clin Exp Med. 2017;10(10):14526‐14531.

[2] Kaya M , Ersoy B , Yeniterzi M . Mitral valve regurgitation due to annular dilatation caused by a huge and floating left atrial myxoma. Kardiochirurgia i Torakochirurgia Polska/Polish Journal of Thoracic and Cardiovascular Surgery. 2015;12(3):248‐258. 26702283 10.5114/kitp.2015.54463 PMC4631919

